# Association Between Preoperative Cerebrovascular High-Risk Status and Long-Term Ischemic Stroke After EVAR

**DOI:** 10.3390/jcm15166484

**Published:** 2026-08-21

**Authors:** Linyao Zhu, Chengxin Weng, Jichun Zhao, Bin Huang, Ding Yuan, Tiehao Wang, Jinting Ge, Huawei Zhang, Jiarong Wang

**Affiliations:** 1 Division of Vascular Surgery, Department of General Surgery, West China Hospital, Sichuan University, Chengdu 610041, China; 2 West China School of Medicine, Sichuan University, Chengdu 610041, China; 3 Huaxi MR Research Center (HMRRC), Functional and Molecular Imaging Key Laboratory of Sichuan Province, Department of Radiology, West China Hospital, Sichuan University, Chengdu 610041, China

**Keywords:** abdominal aortic aneurysm, endovascular procedures, ischemia stroke

## Abstract

**Objective:** To investigate the impact of asymptomatic high-risk status for ischemic stroke on patients with abdominal aortic aneurysm undergoing endovascular aortic repair (EVAR). **Methods:** Eligible patients with abdominal aortic aneurysm who underwent EVAR between January 2011 and December 2021 were enrolled in this retrospective cohort study. Propensity score matching (PSM) was used to balance baseline characteristics between the cerebrovascular high-risk group and the standard control group. The impact of cerebrovascular high-risk status on short- and long-term outcomes was assessed using Cox proportional hazards regression and generalized linear models, with results presented as hazard ratios (HRs), odds ratios (ORs), and corresponding 95% confidence intervals (CIs). **Results:** A total of 1080 patients were included (299 high-risk, 781 standard). During the 13-year follow-up period, ischemic stroke occurred in 45 patients (15.1%) in the high-risk group (HR, 7.01; 95% CI, 4.11–11.94; *p* < 0.001). The high-risk group also had a higher incidence of major adverse cardiovascular and cerebrovascular events (MACCEs), which occurred in 129 patients (43.1%) (HR, 1.78; 95% CI, 1.43–2.24; *p* < 0.001). These findings remained consistent across inverse probability of treatment weighting (IPTW) and propensity score matching combined with multivariable generalized linear model (PSM+MVA-GLM) analyses. **Conclusions:** Although no significant increase in perioperative cerebrovascular adverse events was observed in asymptomatic patients with a preoperative cerebrovascular high-risk status, their worse long-term prognosis appears to be associated with this risk status. This association highlights the need for rigorous cardiovascular and cerebrovascular risk management in this vulnerable population after surgery.

## 1. Introduction

Endovascular aortic repair (EVAR) has become the mainstream approach for managing abdominal aortic aneurysms (AAAs) in clinical practice, as it is characterized by lower incidence of perioperative complications. However, patients with AAA frequently have cardiovascular and cerebrovascular comorbidities and are at an elevated risk of stroke, which can be attributed to the shared pathogenesis and risk factors of vascular diseases. Therefore, adverse cardio-cerebrovascular events have emerged as a decisive factor influencing long-term survival in patients who underwent EVAR [1,2,3].

According to the 2024 European Society for Vascular Surgery (ESVS) guidelines, routine preoperative cerebrovascular screening is not recommended, and prophylactic intervention for asymptomatic carotid disease is also not recommended [4]. However, the direct evidence for this recommendation primarily stems from studies on traditional non-cardiac open surgery, and the stroke risk in that population differs significantly from that in patients with AAA. The cardio-cerebrovascular risk in AAA patients is greater than in those with other conditions, compared to individuals without AAA, patients with AAA have an 1.8-fold increase annual stroke risk and a 2.4-fold higher all-cause mortality [5]. As a result, the benefits and risks associated with preoperative cerebrovascular screening in AAA patients at high risk for cerebrovascular disease remain unclear. Moreover, strong associations have been established between stroke/transient ischemic attack and AAA [6,7], there remains insufficient evidence to evaluate the impact of stroke risk factors on postoperative outcomes in patients undergoing elective EVAR [8].

Therefore, this single-center retrospective study aims to determine whether patients with AAA and asymptomatic high cerebrovascular risk profiles (defined as the absence of current symptoms related to cerebrovascular or carotid artery stenosis, including no adverse cerebrovascular events or dizziness symptoms for at least one month before surgery) experience significantly increased rates of postoperative cerebrovascular events following EVAR.

## 2. Materials and Methods

### 2.1. Study Design

This retrospective cohort study was conducted at a tertiary academic hospital in China. The reporting of this observational study adhered to the Strengthening the Reporting of Observational Studies in Epidemiology (STROBE) guidelines. De-identified data were used for analysis and the study was approved by the Institutional Review Board of West China Hospital. The requirement for patient-informed consent was waived owing to the retrospective data collection.

### 2.2. Study Cohort

A total of 1295 consecutive patients diagnosed with infrarenal AAA (ICD-10: I71.4) between January 2011 and December 2021 were retrospectively identified from the hospital information system. The inclusion criteria were patients with AAA (with or without iliac aneurysm) who received EVAR using a bifurcated stent graft. EVAR was performed in the following situations when anatomically eligible [4]: (1) patients with AAA who have reached the diameter threshold for surgery according to guidelines; (2) patients with iliac aneurysm (>3.5 cm) and small AAA (>3.0 cm). Exclusion criteria were: (1) patients undergoing emergency EVAR for ruptured or impending-rupture AAA, (2) preoperative evaluation indicating urgent requirement for cerebrovascular intervention, (3) patients with a history of symptoms such as dizziness or transient syncope.

The primary exposure was defined as whether patients qualified as high-risk for cerebrovascular adverse events prior to EVAR. Patients meeting at least one of the following criteria were classified into the high-risk group: (1) moderate or greater stenosis (≥50%) in one or more major cervical or cerebral arteries (carotid, vertebral, subclavian, or intracranial arteries) [9,10]; (2) prior stroke [11]; and (3) atrial fibrillation [10,12,13]. The remaining patients who underwent EVAR constituted the standard control group. To justify this composite exposure definition, we conceptualized the “high-risk” cohort based on a shared pathophysiological predisposition rather than a single etiology. While these conditions differ in their primary mechanisms, they represent a unified clinical phenotype of compromised cerebrovascular reserve and impaired cerebral autoregulation. During EVAR procedures, patients are routinely exposed to potential cerebral embolic insults arising from catheter manipulation within a highly atheromatous aortic arch and to transient hemodynamic fluctuations such as intraoperative hypotension during graft deployment and anesthetic induction. Therefore, regardless of whether the underlying origin is vascular or cardiogenic, patients carrying any of these risk factors constitute a clinically high-priority population that is highly susceptible to perioperative stroke. Additionally, integrating these conditions into a single exposure category was statistically necessary to preserve adequate statistical power.

### 2.3. Perioperative Management and Surgical Procedure

All patients were managed according to standardized perioperative protocols. Given the shared atherosclerotic pathophysiology of systemic vascular disease, combined cardiovascular and cerebrovascular screening has been adopted as a routine preoperative assessment for elderly patients at our institution.

Following routine anesthesia, disinfection, and draping, bilateral common femoral arteries were punctured, true lumen access was confirmed, and 5-Fr sheaths were placed. Following systemic heparinization and stab incisions, two Perclose ProGlide devices (Abbott Vascular, Santa Clara, CA, USA) were predeployed per access, and 16-Fr sheaths (Cook Medical, Bloomington, IN, USA) were subsequently inserted. Via the left femoral approach, a Glidewire Advantage guidewire (Terumo Corporation, Tokyo, Japan) and a 5-Fr pigtail catheter (Cordis Corporation, Miami Lakes, FL, USA) were advanced to the level of L1. Through the right femoral artery, an Endurant II main body was deployed with its proximal edge immediately below the origin of the left renal artery, and the contralateral limb was fully opened. From the left side, the contralateral gate was selectively cannulated. After guidewire exchange, an Endurant II iliac limb was placed, bridging the contralateral limb to a position just above the bifurcation of the left common iliac artery. The main body was then fully released from the right side, and an ipsilateral iliac limb was deployed to the level of the right common iliac bifurcation. Completion angiography demonstrated well-positioned and patent endografts with brisk flow in the external and internal iliac arteries, as well as delayed sac opacification. A Reliant balloon catheter (Medtronic, Minneapolis, CA, USA) was sequentially inflated at the distal iliac landing zones, graft junctions, and the proximal neck. Repeat angiography showed markedly reduced sac opacification. All wires and catheters were withdrawn, the preplaced sutures were tightened, and manual compression was applied. Dorsalis pedis pulses were palpable bilaterally. The Endurant II or IIs stent graft (Medtronic, Minneapolis, MN, USA) was used in all patients in this study.

Postoperatively, all patients without contraindications to antiplatelet therapy received dual antiplatelet and lipid-lowering therapy. They were also instructed to strictly quit smoking and alcohol; maintain stringent control of blood pressure, lipid levels, and blood glucose; engage in moderate physical activity; adhere to a regular sleep–wake schedule; and attend regular outpatient follow-up.

### 2.4. Study Outcomes

The primary outcome was ischemic stroke, assessed at three distinct time points: within 30 days, at 6 months, and during long-term follow-up. Ischemic stroke was defined by either of the following criteria: (1) acute onset of focal dysfunction of the brain or retina lasting longer than 24 h, and (2) neurological deficits of any duration with corresponding focal infarction confirmed by neuroimaging (CT or MRI) [10].

Secondary outcomes included major adverse cardiovascular and cerebrovascular events (MACCEs), defined as a composite of myocardial infarction, all-cause stroke, and all-cause mortality [14]. Myocardial infarction was defined as new or presumed new significant ST-T wave changes or new left bundle branch block on ECG; appearance of pathological Q waves; imaging evidence of new loss of viable myocardium or new regional wall motion abnormality; or identification of an intracoronary thrombus by angiography or autopsy. Furthermore, all-cause mortality was evaluated as an independent secondary outcome.

### 2.5. Follow-Up Protocol

Patients in the EVAR cohort were scheduled for standardized ultrasound surveillance at 1 month, 6 months, and 12 months postoperatively, and annually thereafter. The occurrence of postoperative ischemic stroke events was determined based on patients’ symptoms and clinical records, with CT or MRI imaging performed when necessary for confirmation. Outcome events were identified through a combination of the hospital medical record system and regularly scheduled follow-up clinical visits (or telephone interviews). To minimize referral and underreporting bias, outcome adjudication explicitly included events diagnosed both at our study center and at outside medical facilities. For events occurring within our institution, diagnoses were extracted from the electronic database and confirmed by corresponding clinical records and in-house positive imaging findings (e.g., Duplex ultrasound, Computed Tomography Angiography [CTA], or digital subtraction angiography [DSA]). For potential events diagnosed at outside centers, primary medical records, discharge summaries, and external imaging materials (films or reports) were obtained from the patients or their relatives. All candidate events were independently reviewed and validated by two senior vascular surgeons blinded to the study groups; any discrepancies were resolved by consensus with a third senior specialist; any discrepancies were resolved by consensus with a third senior specialist. Patients who declined participation or provided incorrect contact information were considered lost to follow-up and were excluded from the final analysis.

### 2.6. Covariables of Interest

Demographic characteristics include sex, gender, smoking status, and body mass index (BMI). Comorbidities were identified through clinical diagnoses and chart review. The comorbidities considered as covariates are diabetes mellitus, hypertension, coronary artery disease (CAD), chronic heart failure (CHF) and chronic kidney disease (CKD) [15,16,17].

### 2.7. Data Collection and Statistical Analysis

All data in this study were retrospectively collected. To ensure data quality, a standardized case report form (CRF) was used for data collection. Data were double-entered by two independent investigators and cross-checked for discrepancies. For long-term follow-up outcome events, all patients who experienced an outcome event were identified and confirmed based on medical records from our hospital or the local hospital. Additionally, all personnel involved in data collection underwent standardized training prior to study initiation. Categorical variables were summarized using frequencies and percentages. Normally distributed continuous variables were presented as mean ± standard deviation (SD), while non-normally distributed variables were expressed as median (Q1, Q3). Group differences for categorical variables were assessed using Fisher’s exact test or the chi-square test. Student’s *t*-test or the Mann–Whitney U test were employed for comparing continuous variables between groups. Standardized mean differences (SMDs) were calculated to evaluate baseline balance, with SMD > 10% indicating substantial imbalance.

Missing data were handled by multiple imputation. Propensity score matching (PSM) was performed to minimize intergroup baseline differences. Based on age, sex, BMI, smoking status, hypertension, and diabetes, the propensity to be classified into the high-risk group for adverse cerebrovascular events was estimated using a logistic regression model. To further evaluate the robustness of the results, CAD, CHF, and CKD were included as additional covariates in Inverse Probability of Treatment Weighting (IPTW) and multivariable regression to account for residual confounding. PSM was performed using 1:3 nearest-neighbor matching without replacement, with a caliper set at 0.02 of the standard deviation of the logit of the propensity score, thereby creating matched groups with minimal average propensity score distance. Patients who could not be matched were excluded from the analysis. Survival curves were generated by using the Kaplan–Meier method. The impact of high-risk status on short- and long-term outcomes was evaluated using Cox proportional hazards regression and generalized linear models, with results reported as hazard ratios (HRs) or odds ratios (ORs) and their corresponding 95% confidence intervals (CIs). The proportional hazards assumption for all Cox models was systematically tested and verified using Schoenfeld residuals, all of which yielded *p* values > 0.05, indicating no violation of the assumption; detailed results are provided in Supplementary Table S1. All statistical analyses were performed using R Studio Version 5.2.1 (http://www.Rproject.org). A significance level of 0.05 was used for all analyses.

In the subgroup analysis, patients were stratified based on two criteria: (1) the presence or absence of moderate-to-severe arterial stenosis, and (2) a history of cerebrovascular adverse events. The sensitivity analysis primarily included the following five factors: carotid artery stenosis ≥ 70%, subclavian or vertebral artery stenosis ≥ 70%, intracranial artery stenosis ≥ 70%, atrial fibrillation, previous cerebrovascular event.

## 3. Results

**Figure 1:**
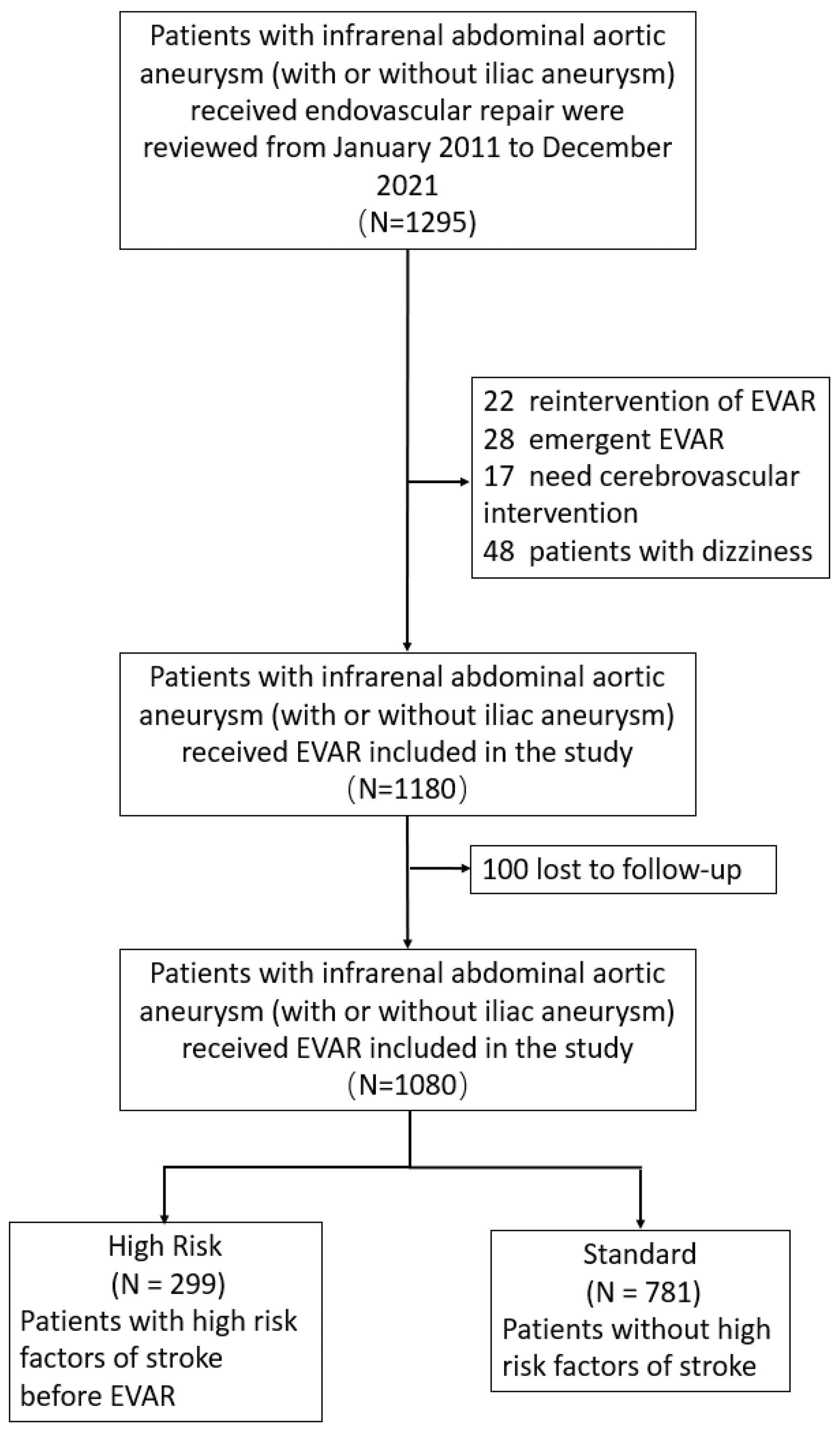
Study flowchart. EVAR, endovascular aortic repair.

A total of 1295 consecutive patients diagnosed with infrarenal AAA were initially identified. Among them, 22 patients requiring re-intervention after EVAR, 28 undergoing emergent surgery for rapid disease progression, 48 with a history of dizziness, and 17 whose preoperative evaluation indicated a need for prior cerebrovascular intervention were excluded. Consequently, 1180 patients were eligible for enrollment. After excluding 100 patients (8.5%) lost to follow-up, the final study cohort comprised 1080 patients, including 299 in the high-risk group and 781 in the standard group (Figure 1).

**Figure 2:**
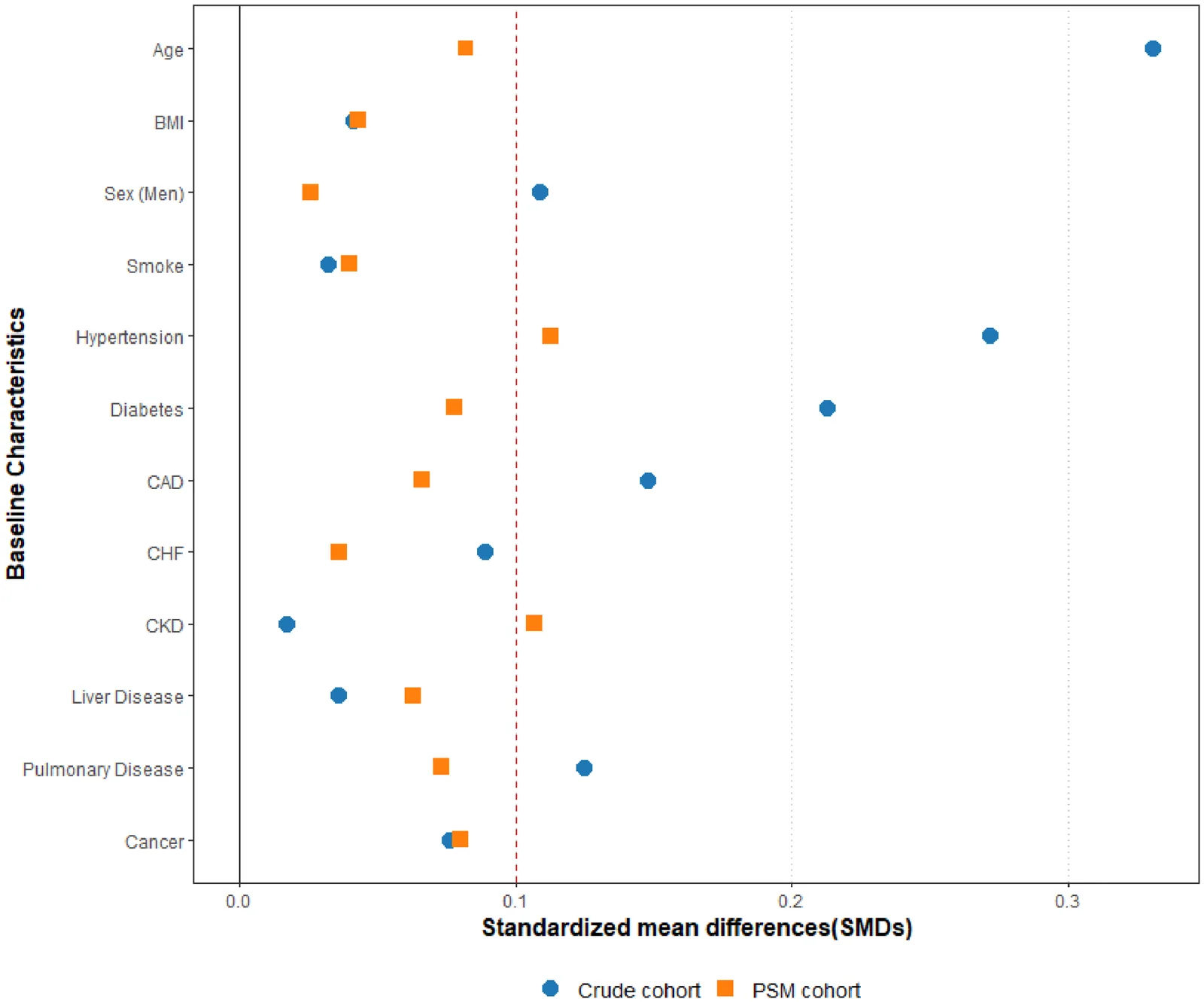
Standardized mean differences of baseline characteristics in crude and PSM cohorts.

Baseline characteristics and high-risk factors before and after PSM are presented respectively in Table 1, Table 2 and Supplementary Table S2. Briefly, patients in the high-risk group were more likely to be older, male, with a history of smoking, and with comorbidities. After PSM, the SMDs for hypertension and chronic kidney disease (CKD) remained slightly above the 0.1 threshold; although the 95% confidence intervals for both variables included zero and the *p*-values exceeded 0.05, the matching was still deemed suboptimal. (Table 2, Figure 2).

### 3.1. Primary Outcomes

#### 3.1.1. 30-Day Ischemic Stroke

All three cerebrovascular adverse events occurring within 30 days postoperatively were observed in the high-risk group both with crude and after PSM (*n* = 3.1% vs. *n* = 0, 0%). In the statistical analyses, employing generalized linear models, no significant association was observed between the high-risk group and the incidence of ischemic stroke following EVAR in crude analysis (*p* = 0.996) and PSM analysis (*p* = 0.997). The results of the Firth penalized logistic regression analysis are presented in Table 3.

In the subgroup analysis, all cases of ischemic stroke occurring within 30 days postoperatively were observed in the high-risk group. Among these, three patients presented with moderate-to-severe (≥50%) vascular stenosis, and one had previous cerebrovascular adverse events. Sensitivity analyses did not identify any significant factors contributing to the incidence of early postoperative stroke during this period.

#### 3.1.2. 6-Month Ischemic Stroke

During the 6-month postoperative period, a total of seven ischemic stroke events were recorded, with six cases (2.0%) occurring in the high-risk group and one case (0.1%) in the standard control group. Following PSM, the incidence rate increased to 2.1% in the high-risk group and 0.2% in the standard control group. Significantly elevated risks were consistently observed across all four analytical models. Consistent results were also observed in the Firth penalized logistic regression analysis (Table 3).

Subgroup analysis revealed that patients with moderate-to-severe arterial stenosis had a significantly increased risk of postoperative ischemic stroke. However, a history of cerebrovascular events was not significantly associated with a rise in the incidence of adverse events. In contrast, sensitivity analyses revealed that these factors were independently associated with an elevated risk of ischemic stroke (Supplementary Figures S1 and S2).

#### 3.1.3. Ischemic Stroke During 13-Year Follow-Up

A total of 66 patients experienced cerebrovascular adverse events during follow-up, with 45 cases (15.1%) in the high-risk group and 21 cases (2.7%) in the standard control group. After PSM, 58 events were analyzed, including 42 (15.0%) in the high-risk group and 16 (2.8%) in the control group.

**Figure 3:**
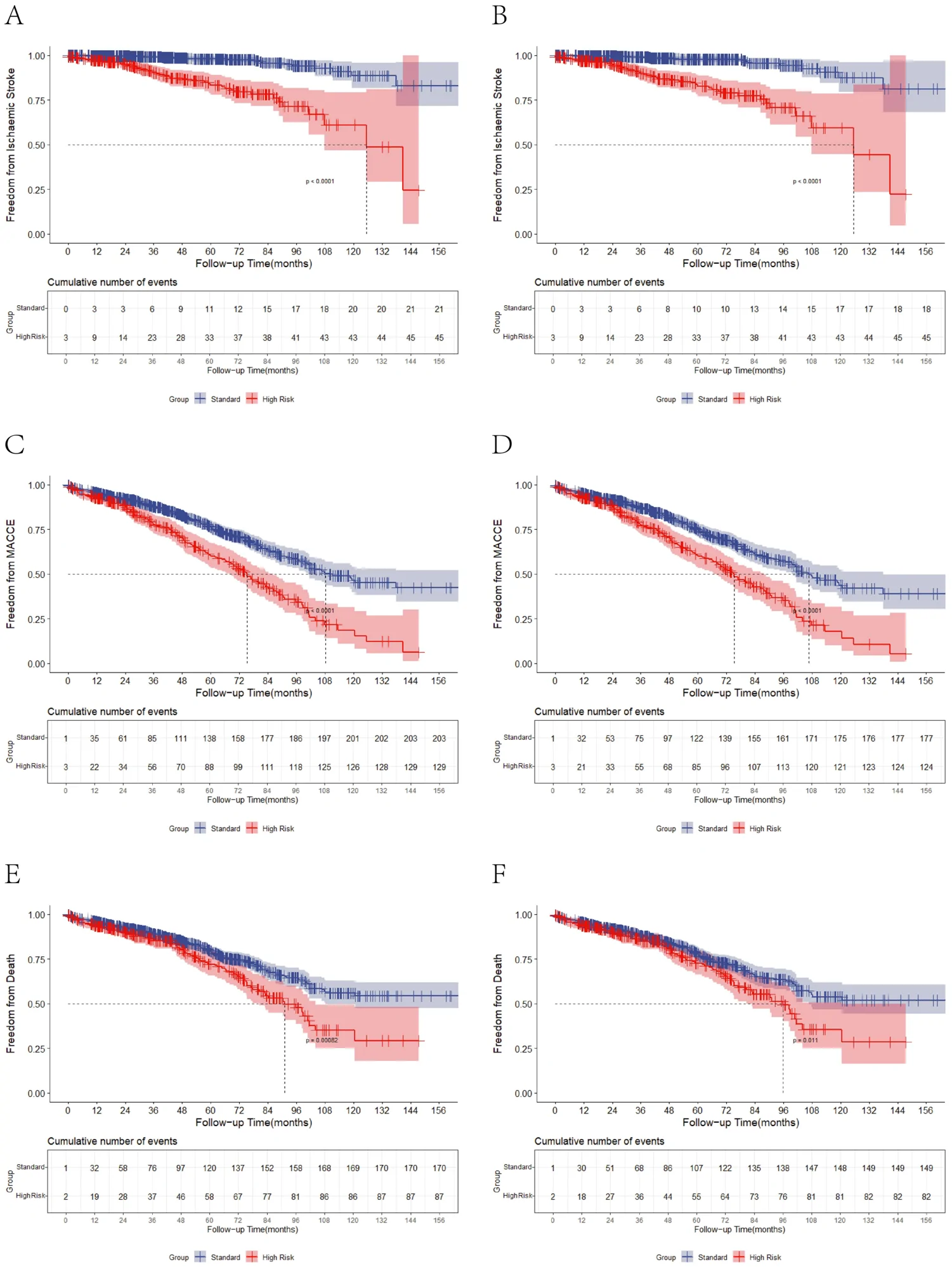
Kaplan–Meier curves illustrate event-free survival from ischemic stroke in crude (**A**) and propensity score-matched (**B**) cohort, MACCEs in crude (**C**) and propensity score-matched (**D**) cohorts, and overall survival in crude (**E**) and propensity score-matched (**F**) cohorts. MACCEs, major adverse cardiac and cerebrovascular events.

Significant differences in ischemic stroke rates were observed between groups in both crude analysis (HR, 7.01; 95% CI, 4.11–11.94; *p* < 0.001) and PSM analysis (HR, 6.88; 95% CI, 3.83–12.33; *p* < 0.001) (Figure 3A,B). These findings were consistently confirmed in IPTW (HR, 7.15; 95% CI, 4.15–12.33; *p* < 0.001) and PSM with multivariable-adjusted Cox regression (PSM+MVA-Cox) (HR, 6.89; 95%CI, 3.84–12.36; *p* < 0.001).

Subgroup analysis showed uniformly increased ischemic stroke risk across all subgroups, with no significant intergroup differences, while sensitivity analysis confirmed that all evaluated factors significantly elevated stroke risk (Supplementary Figures S3 and S4).

### 3.2. Secondary Outcomes

#### 3.2.1. 30-Day Outcomes

##### 30-Day MACCE

A total of 14 MACCE cases occurred within 30 days postoperatively, with 4 (1.3%) in the high-risk group and 10 (1.3%) in the standard control group. After matching, the high-risk group maintained 4 cases (1.4%), while the standard group had 10 cases (1.7%). No significant association was observed between high-risk status and 30-day MACCEs in all analysis models.

In subgroup analyses of the high-risk group, none of the examined subgroups demonstrated a significant increase in the risk of postoperative MACCEs. A similar trend was confirmed in sensitivity analysis (Supplementary Figures S5 and S6).

##### 30-Day All-Cause Mortality

A total of 13 deaths occurred within 30 days postoperatively, comprising 4 (1.3%) in the high-risk group and 9 (1.2%) in the standard control group. After PSM, the absolute numbers remained unchanged, with the mortality rate in the high-risk group and standard control group increasing to 1.4% and 1.6%. No statistically significant differences between groups were observed in all models (Table 3).

In subgroup analysis, patients without vascular stenosis showed a significant increase in postoperative all-cause mortality risk. However, sensitivity analysis did not identify specific factors that independently contributed to this increased risk (Supplementary Figures S7 and S8).

#### 3.2.2. 6-Month Outcomes

##### 6-Month MACCE

A total of 40 MACCEs were documented, with 16 cases (5.4%) occurring in the high-risk group and 24 cases (3.1%) in the standard control group. After PSM, the numbers adjusted to 15 (5.4%) and 18 (3.1%) cases in the high-risk and control groups, respectively. No statistically significant difference in MACCE incidence was observed between the high-risk and control groups in either the crude analysis (OR, 1.58; 95% CI, 0.82–3.06; *p* = 0.175) or the PSM-adjusted analysis (OR, 1.70; 95% CI, 0.84–3.44; *p* = 0.141). Consistent non-significant results were also obtained using IPTW and the combined PSM+MVA-GLM (Table 3).

Subgroup analysis identified stenosis ≥50% as a significant risk factor for postoperative MACCEs, while no similar significant association was observed in the remaining subgroups. Notably, sensitivity analyses identified severe subclavian or vertebral artery stenosis and prior stroke history as significant factors contributing to increased MACCE risk (Supplementary Figures S9 and S10).

##### 6-Month All-Cause Mortality

During the 6-month postoperative period, a total of 37 mortality events were recorded, with 14 cases (4.7%) in the high-risk group and 23 cases (2.9%) in the standard control group. Following PSM, the incidence was 4.6% (13 cases) in the high-risk group and 3.0% (17 cases) in the control group. No statistically significant increase in all-cause mortality was observed in the high-risk group compared to controls in the crude analysis (OR,1.42; 95% CI, 0.71–2.83; *p* = 0.323), the PSM-adjusted analysis (OR, 1.54; 95% CI, 0.73–3.24; *p* = 0.247), or in supplementary analyses using IPTW and PSM+MVA-GLM (Table 3). Subgroup analysis and sensitivity analysis did not identify any factors significantly associated with increased all-cause mortality (Supplementary Figures S11 and S12).

#### 3.2.3. 13-Year Outcomes

##### MACCE During 13-Year Follow-Up

During the follow-up period, 332 MACCE cases were recorded, with 129 (43.1%) in the high-risk group and 203 (26.0%) in the standard control group. After PSM, 280 events were analyzed, comprising 121 (43.2%) in the high-risk group and 159 (27.7%) in the control group. Both crude analysis (HR, 1.78; 95% CI, 1.43–2.24; *p* < 0.001) and PSM analysis (HR, 1.81; 95% CI, 1.42–2.29; *p* < 0.001) demonstrated significantly higher MACCE risk in the high-risk group compared to the standard group (Figure 3C,D). These findings were consistent in IPTW (HR, 1.81; 95%CI, 1.44–2.27; *p* < 0.001) and in PSM+MVA-Cox (HR, 1.83; 95% CI, 1.44–2.32; *p* < 0.001) (Table 3).

All subgroups showed an increased risk trend with no significant intergroup differences, and sensitivity analyses consistently identified these factors as significant predictors of increased MACCE incidence (Supplementary Figures S13 and S14).

##### All-Cause Mortality During 13-Year Follow-Up

Throughout the follow-up period, 257 deaths were recorded, with 87 (29.1%) in the high-risk group and 170 (21.8%) in the standard control group. After propensity score matching (PSM), 215 deaths were analyzed, comprising 81 (28.9%) in the high-risk group and 134 (23.3%) in the control group.

Both crude analysis (HR,1.36; 95% CI, 1.04–1.77; *p* = 0.022) (Figure 3E) and PSM analysis (HR,1.39; 95% CI, 1.05–1.84; *p* = 0.020) (Figure 3F) indicated that high-risk status was associated with increased all-cause mortality. This finding was consistent in IPTW (HR,1.33; 95% CI, 1.02–1.74; *p* = 0.034) and PSM+MVA-Cox (HR,1.41; 95% CI, 1.06–1.86; *p* = 0.016) (Table 3).

In subgroup analysis, vascular stenosis <50% was significantly associated with increased all-cause mortality during follow-up. In sensitivity analysis, neither prior cerebrovascular events nor atrial fibrillation demonstrated a statistically significant association with all-cause mortality (Supplementary Figures S15 and S16).

## 4. Discussion

The relationship between EVAR and asymptomatic high cerebrovascular risk remains inconclusive due to limited and conflicting evidence. In contrast to previous studies, the present study specifically focused on patients with abdominal aortic aneurysm undergoing EVAR and elucidated the association between long-term prognosis and asymptomatic high-risk status for ischemic stroke. Our study demonstrated that asymptomatic patients with high cerebrovascular risk profiles, while showing no significant difference in 30-day outcomes after EVAR, exhibited significantly worse prognosis at both 6 months and during long-term follow-up. These findings warrant a closer examination of the baseline cerebrovascular risk profile inherent to patients with AAA.

First, patients with AAA frequently present with cardiovascular and cerebrovascular diseases due to shared pathogenic mechanisms and risk factors in vascular disease. Evidence indicates that approximately 36% of AAA patients have significant carotid artery disease [18], while 30.4–38% exhibit concomitant carotid artery stenosis (CAS) [8,19]. A total of 10.8% of AAA patients demonstrate severe internal carotid artery stenosis, with a higher prevalence in male patients. According to ESVS guidelines [4,20], at least 8.8% of AAA patients present with asymptomatic carotid stenosis, with an even higher prevalence among those scheduled for EVAR. In addition, some patients present with concurrent subclavian or vertebral artery stenosis, which are risk factors for posterior circulation stroke. Furthermore, the potential presence of intracranial vascular stenosis may further elevate stroke risk. These multiple vascular pathologies may not be adequately identified or integrated in current routine risk assessment frameworks [10,13]. Crucially, the association between asymptomatic cerebrovascular disease and late postoperative stroke extends far beyond a shared atherosclerotic burden. Several interconnected systemic biological mechanisms likely drive this increased long-term risk. Chronic systemic inflammation and endothelial dysfunction create a highly vulnerable vascular environment, promoting thrombus formation on otherwise stable silent lesions, and are drivers of both AAA expansion and remote plaque destabilization [21]. In addition, increased arterial stiffness, which characterizes the diffuse arterial degeneration in these patients, elevates pulse pressure and subjects the cerebral microcirculation to high shear stress, thereby accelerating progressive microvascular remodeling and impairing cerebral autoregulation [22]. Furthermore, subclinical atrial cardiomyopathy, characterized by atrial fibrosis and remodeling, frequently co-exists with advanced systemic atherosclerosis. This condition can promote localized blood stasis and microembolism even in the absence of clinically documented atrial fibrillation [23]. To refine the detection of such cardiac sources of embolism, advanced cardiovascular imaging techniques have recently emerged as critical tools for improving cerebrovascular risk stratification. Specifically, two-dimensional speckle tracking echocardiography (2D-STE) has gained prominence. Increasing evidence indicates that left atrial (LA) reservoir strain is a highly sensitive and early marker of atrial mechanical dysfunction and atrial cardiomyopathy, identifying patients at elevated thromboembolic risk even in the absence of overt atrial fibrillation [24]. Reduced LA reservoir strain has been shown to independently predict adverse outcomes in patients with acute ischemic stroke and can reliably identify individuals harboring an occult cardioembolic substrate who warrant closer clinical surveillance and intensive secondary prevention. Furthermore, recent trials have demonstrated that impaired LA strain parameters are independently associated with postoperative ischemic stroke following major cardiovascular interventions, providing substantial incremental prognostic information beyond conventional indices such as left atrial volume and left ventricular ejection fraction (LVEF) [25]. According to data from our center, approximately 8.1% of patients presented with asymptomatic moderate or greater carotid artery stenosis, which aligns with the current literature. However, after incorporating high-risk factors such as vertebral artery, subclavian artery, and intracranial artery stenosis into a comprehensive risk assessment, a total of 299 patients (27.7% of the cohort) were classified as high-risk. This suggests that the true burden of high-risk cerebrovascular comorbidities in this population is likely underestimated in the existing literature. These findings, coupled with the emerging role of 2D-STE-derived LA strain, highlight a major future opportunity: integrating innovative cardiac imaging with multi-vessel vascular imaging into a comprehensive, multimodal perioperative stroke risk assessment framework. Such a holistic cardiovascular assessment paradigm would significantly improve the identification of high-risk individuals and guide personalized secondary prophylactic strategies. Thus, although EVAR is classified as a non-cardiac surgical procedure, the patient undergoing EVAR carries a higher risk of stroke compared to those undergoing traditional non-cardiac surgery. Our findings demonstrate that, in the GLM analysis, no statistically significant differences were observed between the high-risk and standard control groups in the 30-day ischemic stroke, 6-month MACCEs, or all-cause mortality among asymptomatic patients at high cerebrovascular risk undergoing EVAR. However, the 6-month incidence of ischemic stroke was significantly higher in the high-risk group than in the control group. To further assess the robustness of these results, we performed Firth penalized logistic regression for ischemic stroke events at 30 days and 6 months. The results indicated that, with the exception of the crude model, all other models consistently demonstrated a higher incidence of ischemic stroke in asymptomatic high-risk patients. We attribute this discrepancy primarily to the limited sample size and the low event rates at 30 days and 6 months, which resulted in complete separation and sparse-data bias. Given the extremely small number of perioperative stroke events, the significant results obtained using Firth penalized regression should be interpreted with considerable caution. Although this method reduces small-sample bias, the wide 95% CI still indicates instability of the estimates and limited statistical precision, underscoring the inherent limitations of this perioperative analysis. We also note that the low event rates observed are consistent with current guideline recommendations for this patient population. Over the entire follow-up, the incidences of ischemic stroke, MACCEs, and all-cause mortality were significantly higher in the high-risk group than in the standard control group. Although high-risk patients are inherently susceptible to cerebrovascular events irrespective of surgical intervention, Kaplan–Meier curves revealed a progressive divergence between the two groups over time. This finding suggests that, among patients undergoing EVAR, a preoperative high-risk status is associated with poorer long-term outcomes. Therefore, we recommend that patients identified preoperatively as being at high cerebrovascular risk should receive long-term lifestyle-modification counseling after EVAR to improve the quality of their life.

Second, although a considerable proportion of patients with AAA are at moderate-to-high cerebrovascular risk, the optimal management of these risk factors prior to EVAR remains controversial. Current guidelines and existing studies generally suggest that routine screening for asymptomatic carotid artery stenosis lacks clinical significance for stroke prevention prior to EVAR [20,26], nor is prophylactic intervention for asymptomatic carotid disease recommended [4,26]. However, the studies supporting this conclusion are primarily based on patients undergoing conventional non-cardiac surgeries. Even when vascular surgery patients were included in relevant studies, no dedicated analysis has focused on AAA patients with concomitant high cerebrovascular risk. All these studies have predominantly concentrated on perioperative outcomes and lack comparative data on long-term prognosis. For patients with AAA scheduled for EVAR, neglecting the systemic impact of vascular disease may lead to an underestimation of their cardiovascular and cerebrovascular risks. Therefore, heterogeneity in cohort characteristics and differences in risk stratification methods may limit the generalizability of previous research findings to this specific population [27,28,29,30]. In prior research, the perioperative stroke incidence for EVAR in patients with infrarenal abdominal aortic aneurysm is approximately 0% to 0.3%, which aligns closely with the findings observed in the present study [31,32,33]. Thus, while forgoing the systematic identification of high-risk individuals may be consistent with current guideline recommendations and may maintain relative perioperative safety, a substantial proportion of these patients would miss the opportunity for targeted secondary prevention. Given the inherently high cardiovascular and cerebrovascular risk profile of AAA patients, it is plausible that the omission of such identification is associated with an elevated long-term risk of cardiovascular and cerebrovascular events and, ultimately, poorer long-term prognosis and quality of life [10]. The existing literature supports that stroke significantly aggravates acute kidney injury, postoperative peripheral arterial disease complications, and MACCEs [34,35]. While these findings remain exploratory, they suggest that identifying patients with asymptomatic yet severe cerebrovascular risk factors prior to EVAR might assist in more granular perioperative risk stratification. However, given the retrospective and observational nature of this study, our data do not support a formal recommendation for routine pre-procedural cerebrovascular screening, nor do they demonstrate a direct clinical benefit from such assessments. Notably, in our cohort, the subgroup of high-risk patients who did not undergo targeted preoperative neurological interventions did not exhibit a statistically significant increase in short-term perioperative complications. Nevertheless, their long-term prognosis is highly likely to be governed by systemic cardiovascular and cerebrovascular progression. Consequently, rather than advocating for aggressive pre-EVAR screening, our results underscore the potential value of incorporating standardized, multidisciplinary medical management and structured lifestyle modifications to optimize long-term secondary prevention in this vulnerable population.

Third, medical therapy during follow-up represents a critical confounder that may significantly influence long-term outcomes, including ischemic stroke and MACCEs. In our clinical practice, patients undergoing EVAR receive intraoperative heparin for thrombosis prophylaxis, followed by standardized postoperative anticoagulation with low-molecular-weight heparin and vasoactive/antihypertensive agents during hospitalization. Upon discharge and throughout the long-term follow-up period, guideline-directed medical therapy is actively prescribed, primarily consisting of single antiplatelet therapy (typically aspirin 100 mg/day or clopidogrel 75 mg/day) and moderate-to-high-intensity statins to mitigate systemic cardiovascular risks [10,15,36]. For patients with concomitant atrial fibrillation, oral anticoagulants (e.g., warfarin or novel oral anticoagulants) are prescribed instead of or in combination with antiplatelets. Furthermore, aggressive blood pressure control (targeting a systolic pressure <130 mmHg using angiotensin-converting enzyme inhibitors, beta-blockers, or calcium channel blockers) is routinely recommended to reduce wall stress on the excluded aneurysm sac. However, long-term medication adherence remains a well-recognized challenge. Based on our institutional data, patients undergoing percutaneous EVAR typically return to normal daily activities within 16 days, whereas those undergoing open-cutdown EVAR require approximately 30 days [37]. It is therefore reasonable to infer that beyond 30 days postoperatively, patient adherence to treatment may gradually decline. Multiple prior studies have also confirmed that medication adherence generally decreases over time, and good adherence has been shown to significantly improve long-term prognosis [38,39]. Consequently, the absence of statistically significant differences in ischemic stroke and MACCE rates during the perioperative and early postoperative phases (up to 30 days) may be attributed to the highly controlled, standardized early medical therapy (including anticoagulation and close blood pressure monitoring). In contrast, the subsequent decline in medication adherence and variability in blood pressure control during long-term follow-up could act as unmeasured confounders, potentially contributing to the higher incidence of long-term adverse events. Due to the retrospective nature of this study, detailed longitudinal data on individual medication compliance and home blood pressure fluctuations were not fully captured. This limitation underscores the need for future trials with structured registries to systematically track medication adherence and home blood pressure control, thereby better elucidating their confounding effects on long-term survival.

Fourth, we acknowledge that the individual components of our composite high-risk definition may exert distinct prognostic influences on short- and mid-term clinical outcomes. From a pathophysiological perspective, patients with cervical or intracranial arterial stenosis are predominantly susceptible to hemodynamic-driven hypoperfusion causing watershed infarctions, or to local thromboembolism during acute drops in blood pressure. In contrast, patients with atrial fibrillation are primarily prone to cardiogenic thromboembolism, a risk often exacerbated by the perioperative interruption and resumption of oral anticoagulants. Finally, patients with a history of prior stroke have pre-existing parenchymal brain damage and chronically impaired cerebral autoregulation, rendering them uniquely vulnerable to even mild intraoperative hypoperfusion or microembolization. Although our sample size precluded a fully powered, mutually adjusted multivariable analysis for each individual subgroup, our findings underscore the need for future studies to stratify outcomes by these individual risk components, thereby better delineating their granular prognostic impacts after EVAR. Therefore, further research is required to establish the necessity of precise preoperative prediction and pre-treatment for adverse events in high-risk cerebrovascular patients undergoing EVAR.

## 5. Limitations

The findings of this study should be interpreted within the context of several limitations. First, as a retrospective observational study, it is inherently susceptible to selection and recall biases. Although we reviewed cases over a substantial 10-year period to establish a large clinical cohort, the retrospective single-center design inevitably introduces potential selection bias. Furthermore, despite the use of propensity score matching to reduce baseline imbalances, residual confounding from incompletely balanced covariates (e.g., hypertension and CKD) and unmeasured variables may still have influenced the observed associations. Second, our protocol lacked routine, standardized serial imaging evaluations (e.g., serial Duplex ultrasound, CTA, or MRA) during follow-up to monitor the anatomical progression or regression of carotid or intracranial disease. Consequently, we were unable to directly correlate clinical adverse events with dynamic changes in vascular lesions over time. These considerations warrant a cautious interpretation of the findings. Nonetheless, the high consistency between our crude and propensity-matched analyses supports the robustness and clinical relevance of our observations within this cohort, providing valuable real-world insights.

Second, due to limited sample size, data heterogeneity, and low event rates for ischemic stroke and MACCE at 30 days and 6 months, the analyses of short- and mid-term outcomes—as well as subgroup and sensitivity analyses—were hampered by complete separation and sparse-data bias. Notably, despite PSM, an adequate balance for hypertension and CKD was not achieved. Hypertension and CKD are both potent, independent risk factors for systemic atherogenesis, accelerated vascular calcification, and thromboembolic events, directly confounding cardiovascular and cerebrovascular outcomes. Specifically, residual hypertension may have elevated baseline arterial shear stress and promoted plaque instability, potentially overestimating the risk of ischemic stroke in the cohort with a higher hypertensive burden. Meanwhile, residual CKD—strongly associated with a chronic pro-inflammatory state, media calcification, and heightened susceptibility to contrast-induced acute kidney injury—could have disproportionately driven the incidence of postoperative MACCEs and renal impairment. Although we applied Firth penalized logistic regression to partially mitigate this residual confounding, the effect estimates still lacked precision (reflected by wide confidence intervals), and fitting a highly stable model proved challenging. Consequently, future studies with larger sample sizes and adequate statistical power are warranted to generate more precise effect estimates and to validate these findings.

Third, although some AAA patients are considered as high risk for postoperative silent cerebral infarction, we did not collect cerebral biomarker data; moreover, almost none of the patients underwent brain imaging examinations after EVAR. Consequently, we cannot exclude the possibility of undetected subclinical cerebrovascular events [40].

Fourth, over this 10-year period (2011–2021), the landscape of EVAR has undergone considerable clinical and technological evolution. These advancements include the introduction of lower-profile delivery systems; refined graft fixation mechanisms; the accumulation of institutional and operator experience; and the standardization of perioperative care, such as enhanced recovery pathways and optimized cardioprotective pharmacotherapy. Consequently, these temporal shifts may have introduced unmeasured historical confounding, which could potentially influence early postoperative outcomes and long-term reintervention rates.

## 6. Conclusions

Even among asymptomatic patients, those with high cerebrovascular risk tend to have poorer long-term quality of life after EVAR for AAA. Our findings indicate that these high-risk factors are associated with an elevated risk of stroke during long-term follow-up. Therefore, for asymptomatic patients scheduled for EVAR, enhanced long-term postoperative monitoring of cardiovascular and cerebrovascular risk may help reduce the incidence of ischemic stroke and MACCEs. Larger prospective studies are warranted to validate the robustness of these findings.

## Figures and Tables

**Table 1:** Comparison of baseline characteristics of the crude cohort.

Characteristic	Overall *n* = 1080	Low *n* = 781	High *n* = 299	SMD [95% CI]	*p*-Value ^1^
	Mean (SD)/median [IQR]				
Age (years), Mean (SD)	70.88 (9.70)	70.00 (10.29)	73.18 (7.48)	−0.331 (−0.465–0.197)	<0.001
BMI, Median (IQR)	23.03 (20.76–25.39)	23.03 (20.70–25.39)	23.11 (20.8–25.45)	−0.041 (−0.174, 0.093)	0.476
	Frequent (%)				
Sex, *n* (%)					0.116
Male	897 (83)	640 (82)	257 (86)	−0.109 (−0.24, 0.021)	
Female	183 (17)	141 (18)	42 (14)	0.109 (−0.021, 0.24)	
Smoke, *n* (%)	609 (56)	437 (56)	172 (58)	−0.032 (−0.165, 0.101)	0.641
Hypertension, *n* (%)	695 (64)	475 (61)	220 (74)	−0.274 (−0.405, −0.144)	<0.001
Diabetes, *n* (%)	140 (13)	85 (11)	55 (18)	−0.214 (−0.353, −0.074)	0.001
CAD, *n* (%)	193 (18)	127 (16)	66 (22)	−0.148 (−0.285, −0.011)	0.026
CHF, *n* (%)	28 (2.6)	17 (2.2)	11 (3.7)	−0.089 (−0.23, 0.051)	0.165
CKD, *n* (%)	46 (4.3)	34 (4.4)	12 (4.0)	0.017 (−0.115, 0.149)	0.804
Liver Disease, *n* (%)	18 (1.7)	12 (1.5)	6 (2.0)	−0.036 (−0.173, 0.101)	0.599
Pulmonary Disease, *n* (%)	220 (20)	148 (19)	72 (24)	−0.125 (−0.261, 0.011)	0.061
Cancer, *n* (%)	52 (4.8)	41 (5.2)	11 (3.7)	0.076 (−0.052, 0.204)	0.281

^1^ Wilcoxon rank sum test; Pearson’s Chi-squared test; Fisher’s exact test. Continuous variables are presented as mean (SD) or median [IQR] and categorical variables are presented as frequent (%).

**Table 2:** Comparison of baseline characteristics of the propensity score-matched cohort.

Characteristic	Overall *n* = 855	Low *n* = 575	High *n* = 280	SMD [95% CI]	*p*-Value ^1^
	Mean (SD)/median [IQR]				
Age (years), Mean (SD)	72.47 (7.68)	72.26 (7.85)	72.90 (7.32)	0.082 (−1.705, 0.440)	0.259
BMI, Median (IQR)	22.96 (20.66–25.39)	22.96 (20.48–25.35)	23.00 (20.79–25.39)	0.043 (−0.675, 0.361)	0.422
	Frequent (%)				
Sex, *n* (%)					0.721
Male	735 (86)	496 (86)	239 (85)	0.026 (−0.059, 0.041)	
Female	120 (14)	79 (14)	41 (15)	0.026 (−0.041, 0.059)	
Smoke, *n* (%)	506 (59)	344 (60)	162 (58)	0.040 (−0.051, 0.090)	0.583
Hypertension, *n* (%)	590 (69)	387 (67)	203 (73)	0.113 (−0.117, 0.013)	0.123
Diabetes, *n* (%)	107 (13)	67 (12)	40 (14)	0.078 (−0.075, 0.022)	0.275
CAD, *n* (%)	162 (19)	104 (18)	58 (21)	0.066 (−0.083, 0.031)	0.358
CHF, *n* (%)	24 (2.8)	15 (2.6)	9 (3.2)	0.036 (−0.030, 0.018)	0.615
CKD, *n* (%)	40 (4.7)	31 (5.4)	9 (3.2)	0.107 (−0.006, 0.049)	0.157
Liver Disease, *n* (%)	11 (1.3)	6 (1.0)	5 (1.8)	0.063 (−0.025, 0.010)	0.353
Pulmonary Disease, *n* (%)	190 (22)	122 (21)	68 (24)	0.073 (−0.091, 0.030)	0.311
Cancer, *n* (%)	40 (4.7)	30 (5.2)	10 (3.6)	0.080 (−0.012, 0.045)	0.285

^1^ Wilcoxon rank sum test; Pearson’s Chi-squared test; Fisher’s exact test. Continuous variables are presented as mean (SD) or median [IQR] and categorical variables are presented as frequent (%).

**Table 3:** Comparison of outcomes between high-risk and standard control groups.

High Risk VS. Standard				
**30-Day Outcomes**				
**Models**	High Risk*	Standard*	**OR (95%CI)**	***p***
Ischemic Stroke				
Crude	3/299 (1.0%)	0/781 (0%)	NA	0.996
PSM	3/280 (1.1%)	0/575 (0%)	NA	0.997
IPTW	3/299 (1.0%)	0/781 (0%)	NA	0.995
PSM+MVA-GLM	3/280 (1.1%)	0/575 (0%)	NA	0.997
Ischemic Stroke ^#^				
Crude	3/299 (1.0%)	0/781 (0%)	NA	NA
PSM	3/280 (1.1%)	0/575 (0%)	13.02 (1.29–1738.05)	0.027
IPTW	3/299 (1.0%)	0/781 (0%)	12.08 (1.24–1594.65)	0.030
PSM+MVA-GLM	3/280 (1.1%)	0/575 (0%)	20.52 (2.63–2641.0)	0.001
Major adverse cardiac and cerebrovascular events				
Crude	4/299 (1.3%)	10/781 (1.3%)	0.92 (0.28–3.00)	0.895
PSM	4/280 (1.4%)	10/575 (1.7%)	0.89 (0.27–2.92)	0.843
IPTW	4/299 (1.3%)	10/781 (1.3%)	1.39 (0.75–2.59)	0.298
PSM+MVA-GLM	4/280 (1.4%)	10/575 (1.7%)	0.87 (0.26–2.89)	0.824
Overall mortality				
Crude	4/299 (1.3%)	9/781 (1.2%)	1.16 (0.36–3.81)	0.805
PSM	4/280 (1.4%)	9/575 (1.6%)	0.91 (0.28–2.99)	0.876
IPTW	4/299 (1.3%)	9/781 (1.2%)	1.50 (0.64–3.57)	0.355
PSM+MVA-GLM	4/280 (1.4%)	9/575 (1.6%)	0.89 (0.26–2.97)	0.851
**6-Month Outcomes**				
**Models**	High Risk*	Standard*	**OR (95%CI)**	***p***
Ischemic Stroke				
Crude	6/299 (2.0%)	1/781 (0.1%)	16.24 (1.91–138.12)	0.011
PSM	6/280 (2.1%)	1/575 (0.2%)	12.62 (1.51–105.75)	0.019
IPTW	6/299 (2.0%)	1/781 (0.1%)	18.28 (3.01–111.05)	0.002
PSM+MVA-GLM	6/280 (2.1%)	1/575 (0.2%)	13.00 (1.55–109.24)	0.018
Ischemic Stroke ^#^				
Crude	6/299 (2.0%)	1/781 (0.1%)	11.01 (2.27–106.44)	0.002
PSM	6/280 (2.1%)	1/575 (0.2%)	8.37 (1.82–83.34)	0.005
IPTW	6/299 (2.0%)	1/781 (0.1%)	8.31 (1.76–79.12)	0.006
PSM+MVA-GLM	6/280 (2.1%)	1/575 (0.2%)	13.07 (3.60–95.35)	<0.001
Major adverse cardiac and cerebrovascular events				
Crude	16/299 (5.4%)	24/781 (3.1%)	1.58 (0.82–3.06)	0.175
PSM	15/280 (5.4%)	18/575 (3.1%)	1.70 (0.84–3.44)	0.141
IPTW	16/299 (5.4%)	24/781 (3.1%)	1.48 (0.94–2.31)	0.087
PSM+MVA-GLM	15/280 (5.4%)	18/575 (3.1%)	1.70 (0.83–3.47)	0.150
Overall mortality				
Crude	14/299 (4.7%)	23/781 (2.9%)	1.42 (0.71–2.83)	0.323
PSM	13/280 (4.6%)	17/575 (2.6%)	1.54 (0.73–3.24)	0.247
IPTW	14/299 (4.7%)	23/781 (2.9%)	1.32 (0.83–2.10)	0.243
PSM+MVA-GLM	13/280 (4.6%)	17/575 (2.6%)	1.53 (0.73–3.24)	0.258
**13-year Outcomes**				
**Models**	High Risk*	Standard*	**HR (95%CI)**	***p***
Ischemic Stroke				
Crude	45/299 (15.1%)	21/781 (2.7%)	7.01 (4.11–11.94)	<0.001
PSM	42/280 (15.0%)	16/575 (2.8%)	6.88 (3.83–12.33)	<0.001
IPTW	45/299 (15.1%)	21/781 (2.7%)	7.15 (4.15–12.33)	<0.001
PSM+MVA-Cox	42/280 (15.0%)	16/575 (2.8%)	6.89 (3.84–12.36)	<0.001
Major adverse cardiac and cerebrovascular events				
Crude	129/299 (43.1%)	203/781 (26.0%)	1.78 (1.43–2.24)	<0.001
PSM	121/280 (43.2%)	159/575 (27.7%)	1.81 (1.42–2.29)	<0.001
IPTW	129/299 (43.1%)	203/781 (26.0%)	1.81 (1.44–2.27)	<0.001
PSM+MVA-Cox	121/280 (43.2%)	159/575 (27.7%)	1.83 (1.44–2.32)	<0.001
Overall mortality				
Crude	87/299 (29.1%)	170/781 (21.8%)	1.36 (1.04–1.77)	0.022
PSM	81/280 (28.9%)	134/575 (23.3%)	1.39 (1.05–1.84)	0.020
IPTW	87/299 (29.1%)	170/781 (21.8%)	1.33 (1.02–1.74)	0.034
PSM+MVA-Cox	81/280 (28.9%)	134/575 (23.3%)	1.41 (1.06–1.86)	0.016

High risk*, number of events/number of cohort in high-risk group; Standard*, number of events/number of cohort in standard group; OR, odds ratio; PSM, propensity score matched analysis; PSM+MVA, propensity score matching with multivariable adjustment. ^#^ Firth’s penalized logistic regression.

## Data Availability

The dataset generated and analyzed is available from the corresponding author upon reasonable request.

## Supplementary Materials

The following supporting information can be downloaded at: https://www.mdpi.com/article/10.3390/jcm15166484/s1, Figure S1: Subgroup analyses of 6-month Ischemic Stroke; Figure S2: Sensitivity analyses of 6-month Ischemic Stroke; Figure S3: Subgroup analyses of Ischemic Stroke During 13-year Follow-up; Figure S4: Sensitivity analyses of Ischemic Stroke During 13-year Follow-up; Figure S5: Subgroup analyses of 30-Day MACCE; Figure S6: Sensitivity analyses of 30-Day MACCE; Figure S7: Subgroup analyses of 30-Day All-Cause Mortality; Figure S8: Sensitivity analyses of 30-Day All-Cause Mortality; Figure S9: Subgroup analyses of 6-month MACCE; Figure S10: Sensitivity analyses of 6-month MACCE; Figure S11: Subgroup analyses of 6-month All-Cause Mortality; Figure S12: Sensitivity analyses of 6-month All-Cause Mortality; Figure S13: Subgroup analyses of MACCE During 13-year Follow-up; Figure S14: Sensitivity analyses of MACCE During 13-year Follow-up; Figure S15: Subgroup analyses of All-Cause Mortality During 13-year Follow-up; Figure S16: Sensitivity analyses of All-Cause Mortality During 13-year Follow-up; Table S1. *p* values from proportional hazards assumption using Schoenfeld residuals for the multivariable Cox regression model; Table S2: Comparison of High Risks of the Crude and Propensity Score–Matched Cohort.

## Abbreviations

The following abbreviations are used in this manuscript:

EVAR	Endovascular aortic repair
AAA	abdominal aortic aneurysms
ESVS	European Society for Vascular Surgery
MACCEs	Major adverse cardiovascular and cerebrovascular events
BMI	body mass index
CRF	case report form
SD	standard deviation
SMDs	standardized mean differences
PSM	propensity score matching
CAD	coronary artery disease
CHF	chronic heart failure
CKD	chronic kidney disease
HRs	hazard ratios
ORs	odds ratios
CI	confidence interval
IQR	interquartile range
IPTW	inverse probability of treatment weighting
CTA	computed tomography angiography
DSA	digital subtraction angiography
LA	Left artery
LVEF	left ventricular ejection fraction
PH	proportional hazards
2D-STE	two-dimensional speckle tracking echocardiography

## References

[1] Nicolajsen C.W., Søgaard M., Jensen M., Eldrup N., Larsen T.B., Goldhaber S.Z., Behrendt C.-A., Nielsen P.B. (2023). Antiplatelet Therapy in Patients with Abdominal Aortic Aneurysm Without Symptomatic Atherosclerotic Disease. JAMA Netw. Open.

[2] Bath M.F., Gokani V.J., Sidloff D.A., Jones L.R., Choke E., Sayers R.D., Bown M.J. (2015). Systematic Review of Cardiovascular Disease and Cardiovascular Death in Patients with a Small Abdominal Aortic Aneurysm. Br. J. Surg..

[3] Khashram M., Williman J.A., Hider P.N., Jones G.T., Roake J.A. (2016). Systematic Review and Meta-Analysis of Factors Influencing Survival Following Abdominal Aortic Aneurysm Repair. Eur. J. Vasc. Endovasc. Surg..

[4] Wanhainen A., Van Herzeele I., Bastos Goncalves F., Bellmunt Montoya S., Berard X., Boyle J.R., D’Oria M., Prendes C.F., Karkos C.D., Kazimierczak A. (2024). Editor’s Choice—European Society for Vascular Surgery (ESVS) 2024 Clinical Practice Guidelines on the Management of Abdominal Aorto-Iliac Artery Aneurysms. Eur. J. Vasc. Endovasc. Surg..

[5] Eldrup N., Budtz-Lilly J., Laustsen J., Bibby B.M., Paaske W.P. (2012). Long-Term Incidence of Myocardial Infarct, Stroke, and Mortality in Patients Operated on for Abdominal Aortic Aneurysms. J. Vasc. Surg..

[6] Vänni V., Turtiainen J., Kaustio U., Toivanen J., Rusanen M., Hernesniemi J. (2021). Prospective Ultrasound Screening of Men with Cerebrovascular Disease for Abdominal Aortic Aneurysms. Scand. J. Surg..

[7] Van Lindert N.H.A., Bienfait H.P., Gratama J.W.C., Vriesema H., Ten Hove W., Vermeulen E.G.J., Van Leeuwen R.B. (2009). Screening for Aneurysm of the Abdominal Aorta: Prevalence in Patients with Stroke or TIA. Eur. J. Neurol..

[8] Marsico F., Giugliano G., Ruggiero D., Parente A., Paolillo S., Guercio L.D., Esposito G., Trimarco B., Filardi P.P. (2015). Prevalence and Severity of Asymptomatic Coronary and Carotid Artery Disease in Patients with Abdominal Aortic Aneurysm. Angiology.

[9] Howard D.P.J., Gaziano L., Rothwell P.M. (2021). Risk of Stroke in Relation to Degree of Asymptomatic Carotid Stenosis: A Population-Based Cohort Study, Systematic Review, and Meta-Analysis. Lancet Neurol..

[10] Hankey G.J. (2017). Stroke. Lancet.

[11] Kwok G.Y.R., Chen R.W.R., Leow T.A., Kok C., Yeong N., Teo Y.-H., Low C.E., Wright S., Fink J., Sharma V.K. (2026). Recurrent Ischemic Stroke in Young Adults: A Multicenter Cohort Study, Systematic Review, and Meta-Analysis. Int. J. Stroke.

[12] Nicolajsen C.W., Nielsen P.B., Jensen M., Eldrup N., Larsen T.B., Lip G.Y.H., Goldhaber S.Z., Søgaard M. (2023). Stroke and Myocardial Infarction in Patients with Abdominal Aortic Aneurysm and New-Onset Atrial Fibrillation. Thromb. Haemost..

[13] Gutierrez J., Turan T.N., Hoh B.L., Chimowitz M.I. (2022). Intracranial Atherosclerotic Stenosis: Risk Factors, Diagnosis, and Treatment. Lancet Neurol..

[14] Ueda P., Jernberg T., James S., Alfredsson J., Erlinge D., Omerovic E., Persson J., Ravn-Fischer A., Tornvall P., Svennblad B. (2018). External Validation of the DAPT Score in a Nationwide Population. J. Am. Coll. Cardiol..

[15] Bushnell C., Kernan W.N., Sharrief A.Z., Chaturvedi S., Cole J.W., Cornwell W.K., Cosby-Gaither C., Doyle S., Goldstein L.B., Lennon O. (2024). 2024 Guideline for the Primary Prevention of Stroke: A Guideline from the American Heart Association/American Stroke Association. Stroke.

[16] Gratama J.W.C., Van Leeuwen R.B. (2010). Abdominal Aortic Aneurysm: High Prevalence in Men over 59 Years of Age with TIA or Stroke, a Perspective. Abdom. Imaging.

[17] Liu L., Li Z., Zhou H., Duan W., Huo X., Xu W., Li S., Nie X., Liu H., Liu J. (2023). Chinese Stroke Association Guidelines for Clinical Management of Ischaemic Cerebrovascular Diseases: Executive Summary and 2023 Update. Stroke Vasc. Neurol..

[18] Vranes M., Davidovic L., Vasic D., Radmili O. (2013). Coexistence of Internal Carotid Artery Stenosis in Patients with Abdominal Aortic Aneurysm. Korean Circ. J..

[19] Paraskevas K.I., Nordon I.M., Baxter S.J., Shearman C.P., Phillips M.J. (2016). Abdominal Aortic Aneurysms, Peripheral Arterial Disease, and Carotid Artery Stenosis: Different Sides of the Same Coin?. Angiology.

[20] Wanhainen A., Verzini F., Van Herzeele I., Allaire E., Bown M., Cohnert T., Dick F., Van Herwaarden J., Karkos C., Koelemay M. (2019). Editor’s Choice—European Society for Vascular Surgery (ESVS) 2019 Clinical Practice Guidelines on the Management of Abdominal Aorto-Iliac Artery Aneurysms. Eur. J. Vasc. Endovasc. Surg..

[21] Libby P. (2021). The Changing Landscape of Atherosclerosis. Nature.

[22] Laurent S., Cockcroft J., Van Bortel L., Boutouyrie P., Giannattasio C., Hayoz D., Pannier B., Vlachopoulos C., Wilkinson I., Struijker-Boudier H. (2006). Expert Consensus Document on Arterial Stiffness: Methodological Issues and Clinical Applications. Eur. Heart J..

[23] Martignani C., Spadotto A., Carelli M., Massaro G., Bartoli L., Diemberger I., Biffi M., Corsi C., Zanuttigh B. (2025). Atrial Cardiomyopathy: A “Distinct Clinical Entity” for a Deeper Understanding of Atrial Fibrillation and Cardioembolic Stroke. JCM.

[24] Sonaglioni A., Cara M.D., Nicolosi G.L., Eusebio A., Bordonali M., Santalucia P., Lombardo M. (2021). Rapid Risk Stratification of Acute Ischemic Stroke Patients in the Emergency Department: The Incremental Prognostic Role of Left Atrial Reservoir Strain. J. Stroke Cerebrovasc. Dis..

[25] Vyff F., Johansen N.D., Olsen F.J., Duus L.S., Lindberg S., Fritz-Hansen T., Pedersen S., Iversen A., Galatius S., Møgelvang R. (2023). Left Atrial Reservoir Strain Predicts Ischaemic Stroke after Coronary Artery Bypass Grafting Independent of Postoperative Atrial Fibrillation. Eur. Heart J. Open.

[26] Naylor A.R., Ricco J.-B., De Borst G.J., Debus S., De Haro J., Halliday A., Hamilton G., Kakisis J., Kakkos S., Lepidi S. (2018). Editor’s Choice—Management of Atherosclerotic Carotid and Vertebral Artery Disease: 2017 Clinical Practice Guidelines of the European Society for Vascular Surgery (ESVS). Eur. J. Vasc. Endovasc. Surg..

[27] Sonny A., Gornik H.L., Yang D., Mascha E.J., Sessler D.I. (2014). Lack of Association between Carotid Artery Stenosis and Stroke or Myocardial Injury after Noncardiac Surgery in High-Risk Patients. Anesthesiology.

[28] Ballotta E., Renon L., Da Giau G., Barbon B., De Rossi A., Baracchini C. (2005). Prospective Randomized Study on Asymptomatic Severe Carotid Stenosis and Perioperative Stroke Risk in Patients Undergoing Major Vascular Surgery: Prophylactic or Deferred Carotid Endarterectomy?. Ann. Vasc. Surg..

[29] Axelrod D.A., Stanley J.C., Upchurch G.R., Khuri S., Daley J., Henderson W., Demonner S., Henke P.K. (2004). Risk for Stroke after Elective Noncarotid Vascular Surgery. J. Vasc. Surg..

[30] Sharifpour M., Moore L.E., Shanks A.M., Didier T.J., Kheterpal S., Mashour G.A. (2013). Incidence, Predictors, and Outcomes of Perioperative Stroke in Noncarotid Major Vascular Surgery. Anesth. Analg..

[31] Verhoeven E.L.G., Katsargyris A., Bachoo P., Larzon T., Fisher R., Ettles D., Boyle J.R., Brunkwall J., Böckler D., Florek H.-J. (2014). Real-World Performance of the New C3 Gore Excluder Stent-Graft: 1-Year Results from the European C3 Module of the Global Registry for Endovascular Aortic Treatment (GREAT). Eur. J. Vasc. Endovasc. Surg..

[32] Swerdlow N.J., Liang P., Li C., Dansey K., O’Donnell T.F.X., De Guerre L.E.V.M., Varkevisser R.R.B., Patel V.I., Wang G.J., Schermerhorn M.L. (2020). Stroke Rate after Endovascular Aortic Interventions in the Society for Vascular Surgery Vascular Quality Initiative. J. Vasc. Surg..

[33] Patel P.B., Marcaccio C.L., Swerdlow N.J., O’Donnell T.F.X., Rastogi V., Marino R., Patel V.I., Zettervall S.L., Lindsay T., Schermerhorn M.L. (2023). Thoracoabdominal Aortic Aneurysm Life-Altering Events Following Endovascular Aortic Repair in the Vascular Quality Initiative. J. Vasc. Surg..

[34] Lee O.-H., Ko Y.-G., Ahn C.-M., Shin D.-H., Kim J.-S., Kim B.-K., Choi D., Lee D.Y., Hong M.-K., Jang Y. (2018). Peripheral Artery Disease Is Associated with Poor Clinical Outcome in Patients with Abdominal Aortic Aneurysm after Endovascular Aneurysm Repair. Int. J. Cardiol..

[35] Lentini P., Zanoli L., Fatuzzo P., Husain-Syed F., Stramanà R., Cognolato D., Catena V., Baiocchi M., Granata A., Dell’Aquila R. (2018). Stroke Volume Variation and Serum Creatinine Changes during Abdominal Aortic Aneurysm Surgery: A Time-Integrated Analysis. J. Nephrol..

[36] Chang R.W., Tucker L.-Y., Rothenberg K.A., Lancaster E., Faruqi R.M., Kuang H.C., Flint A.C., Avins A.L., Nguyen-Huynh M.N. (2022). Incidence of Ischemic Stroke in Patients with Asymptomatic Severe Carotid Stenosis Without Surgical Intervention. JAMA.

[37] Zhou Y., Wang J., Zhao J., Yuan D., Weng C., Huang B., Wang T. (2025). Comparison of Percutaneous versus Cutdown Access in Patients after Endovascular Abdominal Aortic Repair: A Randomized Controlled Trial (SWEET-EVAR Trial). Int. J. Surg..

[38] Rymer J.A., Fonseca E., Bhandary D.D., Kumar D., Khan N.D., Wang T.Y. (2021). Difference in Medication Adherence between Patients Prescribed a 30-day versus 90-day Supply after Acute Myocardial Infarction. JAHA.

[39] Rasmussen J.N., Chong A., Alter D.A. (2007). Relationship between Adherence to Evidence-Based Pharmacotherapy and Long-Term Mortality after Acute Myocardial Infarction. JAMA.

[40] Giele J.L.P., Witkamp T.D., Mali W.P.T.M., Van Der Graaf Y. (2004). Silent Brain Infarcts in Patients with Manifest Vascular Disease. Stroke.

