# Supplementary material for: Association Between Preoperative Cerebrovascular High-Risk Status and Long-Term Ischemic Stroke After EVAR
Source: J Clin Med. 2026 Aug 21;15(16):6484. doi: 10.3390/jcm15166484 (PMC13514172; doi:10.3390/jcm15166484)
Supplement: Supplementary file 1 [file jcm-15-06484-s001.zip › jcm-4470988-supplementary.pdf]

Association between preoperative cerebrovascular high-risk status and long-term ischemic stroke after EVAR

Supplementary Online Content

**Supplemental Tables**	
Table S1. P values from proportional hazards assumption using Schoenfeld residuals for the multivariable Cox regression model.	2
Table S2: Comparison of High Risks of the Crude and Propensity Score–Matched Cohort.	2
**Supplemental Figures**	
Figure S1: Subgroup analyses of 6-month Ischemic Stroke	3
Figure S2: Sensitivity analyses of 6-month Ischemic Stroke	3
Figure S3: Subgroup analyses of Ischemic Stroke During 13-year Follow-up	3
Figure S4: Sensitivity analyses of Ischemic Stroke During 13-year Follow-up	4
Figure S5: Subgroup analyses of 30-Day MACCE	4
Figure S6: Sensitivity analyses of 30-Day MACCE	4
Figure S7: Subgroup analyses of 30-Day All-Cause Mortality	5
Figure S8: Sensitivity analyses of 30-Day All-Cause Mortality	5
Figure S9: Subgroup analyses of 6-month MACCE	5
Figure S10: Sensitivity analyses of 6-month MACCE	6
Figure S11: Subgroup analyses of 6-month All-Cause Mortality	6
Figure S12: Sensitivity analyses of 6-month All-Cause Mortality	6
Figure S13: Subgroup analyses of MACCE During 13-year Follow-up	7
Figure S14: Sensitivity analyses of MACCE During 13-year Follow-up	7
Figure S15: Subgroup analyses of All-Cause Mortality During 13-year Follow-up	7
Figure S16: Sensitivity analyses of All-Cause Mortality During 13-year Follow-up	8

	Ischemic Stroke		MACCE		Overall mortality	
**Variable**	**Crude**	**PSM**	**Crude**	**PSM**	**Crude**	**PSM**
High-risk group	0.551	0.670	0.091	0.079	0.118	0.093
Age	0.349	0.266	0.108	0.077	0.060	0.076
Sex	0.720	0.962	0.221	0.227	0.154	0.131
BMI	0.540	0.428	0.099	0.127	0.066	0.059
Smoke	0.657	0.519	0.719	0.829	0.957	0.709
Hypertension	0.341	0.370	0.849	0.832	0.944	0.733
Diabetes	0.733	0.966	0.529	0.368	0.268	0.204
coronary artery disease	0.927	0.841	0.165	0.219	0.073	0.153
chronic heart failure	0.611	0.326	0.996	0.901	0.784	0.369
chronic kidney disease	0.641	0.669	0.599	0.654	0.547	0.576
Global	0.971	0.926	0.170	0.215	0.091	0.095

Table S1: P values from proportional hazards assumption using Schoenfeld residuals for the multivariable Cox regression model.

	Crude		PSM	
**Characteristic**	**N**	**Frequency (%)**	**N**	**Frequency (%)**
Carotid artery stenosis 50%-70%	56	18.7	52	18.6
Carotid artery stenosis ≥70%	31	10.4	29	10.4
Vertebral artery stenosis 50%-70%	22	7.3	20	7.1
Vertebral artery stenosis≥70%	32	10.7	28	10.0
Subclavian artery stenosis 50%-70%	11	3.7	11	3.9
Subclavian artery stenosis≥70%	9	3.0	7	2.5
Intracranial artery stenosis 50%-70%	10	3.3	10	3.6
Intracranial artery stenosis≥70%	7	2.3	6	2.1
Previous cerebrovascular events	171	57.1	163	58.2
Atrial fibrillation	19	6.4	17	6.1

Table S2: Comparison of High Risks of the Crude and Propensity Score–Matched Cohort.

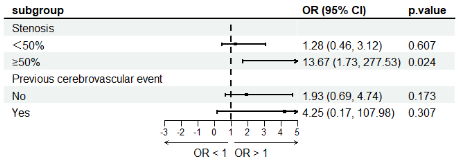

Figure S1：Subgroup analyses of 6-month Ischemic Stroke

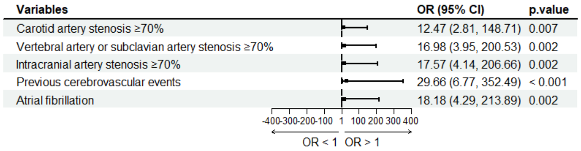

Figure S2：Sensitivity analyses of 6-month Ischemic Stroke

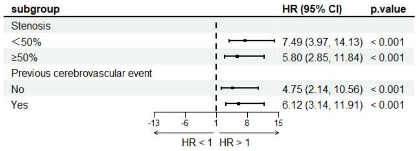

Figure S3：Subgroup analyses of Ischemic Stroke During 13-year Follow-up

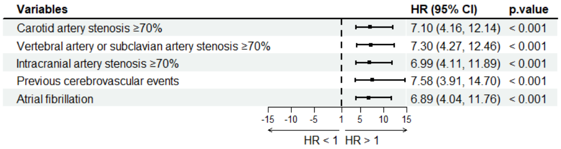

Figure S4： Sensitivity analyses of Ischemic Stroke During 13-year Follow-up

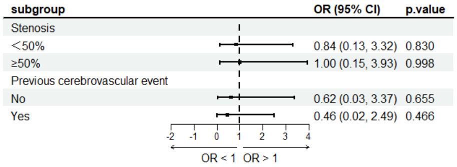

Figure S5： Subgroup analyses of 30-Day MACCE

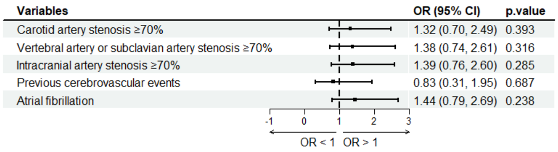

Figure S6：Sensitivity analyses of 30-Day MACCE

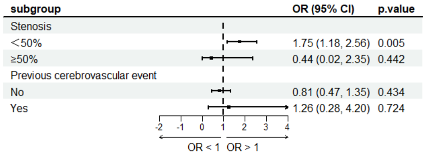

Figure S7：Subgroup analyses of 30-Day All-Cause Mortality

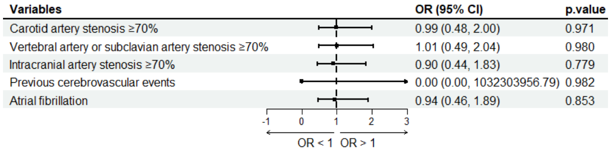

Figure S8：Sensitivity analyses of 30-Day All-Cause Mortality

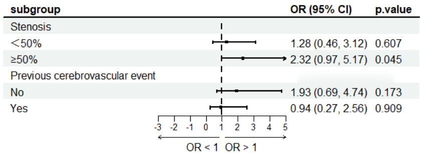

Figure S9：Subgroup analyses of 6-month MACCE

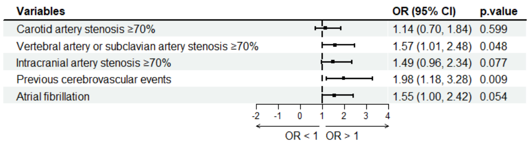

Figure S10：Sensitivity analyses of 6-month MACCE

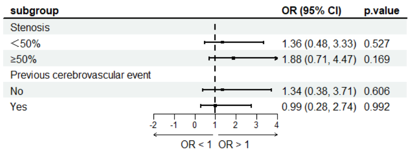

Figure S11：Subgroup analyses of 6-month All-Cause Mortality

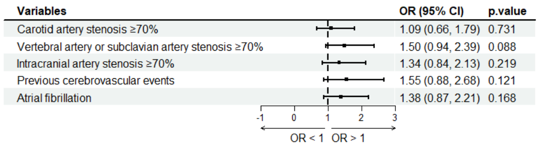

Figure S12：Sensitivity analyses of 6-month All-Cause Mortality

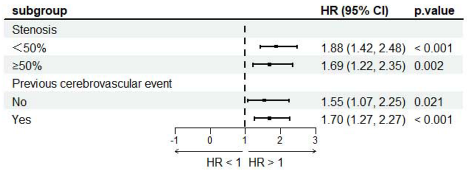

Figure S13：Subgroup analyses of MACCE During 13-year Follow-up

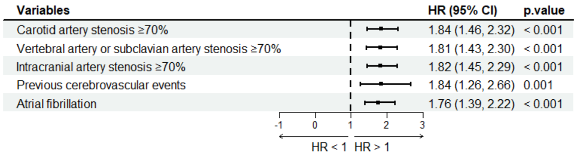

Figure S14：Sensitivity analyses of MACCE During 13-year Follow-up

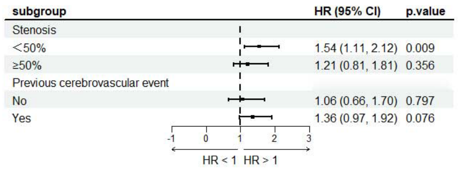

Figure S15：Subgroup analyses of All-Cause Mortality During 13-year Follow-up

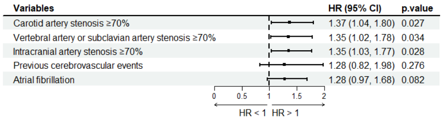

Figure S16：Sensitivity analyses of All-Cause Mortality During 13-year Follow-up
